# Congenital Malaria due to Plasmodium Vivax Infection in a Neonate

**DOI:** 10.1155/2016/1929046

**Published:** 2016-08-29

**Authors:** Ravi Bhatia, Dinesh Rajwaniya, Priti Agrawal

**Affiliations:** ^1^Department of Pediatrics, Pacific Medical College and Hospital, Udaipur, India; ^2^Department of Pathology, Pacific Medical College and Hospital, Udaipur, India

## Abstract

Although malaria is endemic in India, congenital malaria is not very common. Congenital malaria is a very rare condition in both endemic and nonendemic areas. We report a case of congenital malaria in a six-day-old neonate with fever and splenomegaly. The diagnosis was picked up accidentally on a peripheral smear examination. Congenital malaria should be kept as differential diagnosis of neonatal sepsis. Timely detection of this condition could lead to early diagnosis and treatment, thereby preventing neonatal mortality.

## 1. Introduction

Congenital malaria is defined as malarial parasite demonstrated in the peripheral smear of a newborn from 24 hours to seven days of life. Clinically apparent congenital malaria is rare in countries where malaria is endemic and levels of maternal antibodies are high. With the symptoms of congenital malaria being nonspecific, it is often confused with neonatal sepsis.

## 2. Case Representation

A six-day-old male neonate was brought to our outpatient department with complaints of not accepting feed, fever, and loose stools since two days. On examination the baby was pale, icteric, and lethargic, axillary temp was 100°F, and liver and spleen were palpable 3 and 6 cm below the right and left subcostal margins, respectively. Other systems examinations were normal. A provisional diagnosis of neonatal sepsis was made and the patient was started on intravenous ampicillin and gentamycin. Complete blood count (CBC) at the time of admission revealed a Hb of 9.0 gm/dL, total leucocyte count of 10,000 cells/cu mm, differential count of 50% polymorphs, 47% leucocytes, 3% monocytes, and a platelet count of 50,000 cells/cu mm. Other relevant results included a total bilirubin of 10 mg/dL, indirect fraction of 9 mg/dL, Serum Glutamate Pyruvate Transaminase (SGPT) of 485 IU/L, and C-reactive protein (CRP) of 23 mg/dL (*n* = 0–6 mg/dL). Blood, urine, and cerebrospinal fluid cultures were sterile. Chest X-ray was unremarkable. The peripheral blood film revealed trophozoites and schizonts of* P. vivax*, with a parasite index of 2%. The baby was given chloroquine base at dose of 10 mg/kg stat followed by 5 mg/kg at 6, 24, and 48 hrs. There was prompt relief in fever and the spleen gradually reduced in size over a week. Five days after treatment, the patient's parasitemia completely cleared, TLC increased to 12000 cells/cu mm, platelet count was 150,000 cells/cu mm, and CRP fell to 5.5 mg/dL. The infant was discharged on day 5 and is doing well on follow-up.

In view of the revised diagnosis, the history of the mother was reevaluated; a history of fever with chills could be elicited in the ninth month of pregnancy; however presently she did not have any fever. Her peripheral blood film was negative for any malarial parasite. Optimal test was also negative for both* P. falciparum* and* P. vivax*.

## 3. Discussion

Congenital malaria is defined as presence of asexual stages of parasite in cord blood at time of delivery or in the peripheral smear of neonate in the first seven days of life [1]. While* P. falciparum* has been reported more often as a cause of congenital malaria,* P. vivax* as a cause of congenital malaria has been described from southeast Asian region [2].* P. vivax* is the leading cause of congenital malaria in Europe while* P. falciparum* remains the leading cause in Indian and African subcontinent. Neonatal malaria is rare with occurrence rate of 0.3% in immune mothers and 7.4% in nonimmune mothers [3–5]. Placental infection occurs in as many as one-third of women who acquire the infection during pregnancy. The spontaneous clearance of infection in neonates in endemic areas may be as high as 93%. This is attributed to the protective effect of maternal antibodies and role of fetal hemoglobin in slowing the rate of parasite development. Since malaria is thought to be rare in neonates, most cases are accidently picked up on peripheral blood examination as a part of routine sepsis work-up.

Though clinical features of congenital malaria are often nonspecific, presence of fever, anemia, and splenomegaly is a pointer towards congenital malaria. This case shows the importance of considering congenital malaria as a differential diagnosis of neonatal sepsis in neonates born to mothers in malarial endemic countries or with a history of malaria during pregnancy.

Postulated mechanisms for congenital transmission of malaria include maternal transfusion into fetal circulation at the time of delivery or during pregnancy, direct penetration through chorionic villi, or penetration via premature rupture of placenta. The remarkable capacity of fetus to resist malarial infection has been demonstrated. Presence of placental barrier, transfer of protective maternal antibodies, and high levels of fetal hemoglobin are all thought to be protective factors. Congenital malaria can occur despite the absence of any evidence of active malarial infection in mother during pregnancy. It is speculated that the mother had an episode of malaria during the ninth month of pregnancy which was mild, resolved spontaneously, and remained undiagnosed. The lack of maternal parasitemia and HRP2 (histidine rich parasite) antigenemia suggests that the infection was localized to placenta and had cleared. Discordance between maternal peripheral blood examination/antigen testing and placental parasitization is well described. The time of onset of symptoms in congenital malaria can vary from immediately after birth to few weeks though the median age of manifestation has been described as 21 days.

More than 150 cases of congenital malaria have been reported in world literature [6–10]. Congenital malaria presenting as neonatal sepsis and respiratory distress has been described in few reports from across the world [7–10].

The drug of choice for congenital malaria remains chloroquine. The therapy for the infecting plasmodium species is curative. As the infection is produced by transmission of infected erythrocytes rather than forms that invade the liver, the neonate does not require the treatment for exoerythrocytic stages of the parasite.

Our case was accidentally picked up on peripheral blood film examination. This stresses the importance of a good peripheral blood film as a part of all suspected cases of neonatal sepsis. To conclude, congenital malaria is not rare as it was thought to be; in endemic zones malaria should be suspected in all neonates who present with fever and splenomegaly. Early diagnosis could prevent unnecessary antibiotics usage and could prevent neonatal mortality.
